# Characteristics and Clinical Outcome of Breast Cancer Patients with Asymptomatic Brain Metastases

**DOI:** 10.3390/cancers12102787

**Published:** 2020-09-28

**Authors:** Elena Laakmann, Isabell Witzel, Tanja Neunhöffer, Rudolf Weide, Marcus Schmidt, Tjoung-Won Park-Simon, Volker Möbus, Christoph Mundhenke, Arkadius Polasik, Kristina Lübbe, Tobias Hesse, Kerstin Riecke, Marc Thill, Peter A. Fasching, Carsten Denkert, Tanja Fehm, Valentina Nekljudova, Julia Rey, Sibylle Loibl, Volkmar Müller

**Affiliations:** 1 Department of Gynecology, Martinistraße 52, University Medical Center Hamburg-Eppendorf, 20246 Hamburg, Germany; e.laakmann@uke.de (E.L.); iwitzel@uke.de (I.W.); k.riecke@uke.de (K.R.); 2 Frauenärzte am Dom, Rheinstraße 33, 55116 Mainz, Germany; info@frauenaerzte-am-dom.de; 3 Oncological Outpatient Department, Neversstraße 5, 56068 Koblenz, Germany; weide@onkologie-koblenz.de; 4 Department of Gynecology, University Medical Center of the Johannes Gutenberg University Mainz, Langenbeckstr. 1, 55131 Mainz, Germany; Marcus.Schmidt@unimedizin-mainz.de; 5 Hannover Medical School, Department of Gynecology, Carl-Neuberg-Straße 1, 30625 Hannover, Germany; Park-Simon.Tjoung-Won@mh-hannover.de; 6 Department of Hematology and Oncology, University Hospital Frankfurt am Main, Theodor-Stern-Kai 7, 60590 Frankfurt am Main, Germany; Volker.Moebus@kgu.de; 7 Department of Gynecology, Clinic Bayreuth, Preuschwitzer Str. 101, 95445 Bayreuth, Germany; christoph.mundhenke@klinikum-Bayreuth.de; 8 Department of Gynecology, University Medical Center Schleswig-Holstein, Arnold-Heller-Straße 3, 24105 Kiel, Germany; 9 Department of Gynecology and Obstetrics, University Medical Center Ulm, Prittwitzstr. 43, 89075 Ulm, Germany; arkadius.polasik@uniklinik-ulm.de; 10 Diakovere Henriettenstift, Breast Center, Schwemannstraße 17, 30559 Hannover, Germany; Kristina.Luebbe@diakovere.de; 11 Agaplesion Diakonie Clinic Rotenburg, Department of Gynecology, Elise-Averdieck-Straße 17, 27356 Rotenburg, Germany; Hesse@diako-online.de; 12 Department of Gynecology and Gynecological Oncology, Agaplesion Markus Hospital, Wilhelm-Epstein-Str. 4, 60341 Frankfurt am Main, Germany; Marc.Thill@fdk.info; 13 University Hospital Erlangen, Comprehensive Cancer Center Erlangen-EMN, Department of Gynecology and Obstetrics, Friedrich-Alexander University Erlangen-Nuremberg, Universitätsstraße 21-23, 91054 Erlangen, Germany; Peter.Fasching@uk-erlangen.de; 14 Institute of Pathology, University Hospital Marburg, Baldingerstraße, 35043 Marburg, Germany; carsten.denkert@uni-marburg.de; 15 Department of Gynecology, University Hospital Düsseldorf, Moorenstr. 5, 40225 Düsseldorf, Germany; Tanja.Fehm@med.uni-duesseldorf.de; 16 German Breast Group, Martin-Behaim-Straße 12, 63263 Neu-Isenburg, Germany; Valentina.Nekljudova@gbg.de (V.N.); Julia.Rey@gbg.de (J.R.); Sibylle.Loibl@gbg.de (S.L.)

**Keywords:** brain metastases, asymptomatic, breast cancer

## Abstract

**Simple Summary:**

The prognosis for patients with breast cancer that has spread to the brain is poor, and survival for these women hasn’t improved over the last few decades. We do not currently test for asymptomatic brain metastases in breast cancer patients, although this does happen in some other types of cancer. In this study we wanted to find out more about breast cancer that has spread to the brain and in particular to see whether there might be any advantage to spotting brain metastases before the development of neurological symptoms. Overall, our results suggest that women could be better off if their brain metastases are diagnosed before they begin to cause symptoms. We now need to carry out a clinical trial to see what happens if we screen high-risk breast cancer patients for brain metastases. This will verify whether doing so could increase survival, symptom control or quality of life.

**Abstract:**

*Background*: Brain metastases (BM) have become a major challenge in patients with metastatic breast cancer. *Methods*: The aim of this analysis was to characterize patients with asymptomatic BM (*n* = 580) in the overall cohort of 2589 patients with BM from our Brain Metastases in Breast Cancer Network Germany (BMBC) registry. *Results*: Compared to symptomatic patients, asymptomatic patients were slightly younger at diagnosis (median age: 55.5 vs. 57.0 years, *p* = 0.01), had a better performance status at diagnosis (Karnofsky index 80–100%: 68.4% vs. 57%, *p* < 0.001), a lower number of BM (>1 BM: 56% vs. 70%, *p* = 0.027), and a slightly smaller diameter of BM (median: 1.5 vs. 2.2 cm, *p* < 0.001). Asymptomatic patients were more likely to have extracranial metastases (86.7% vs. 81.5%, *p* = 0.003) but were less likely to have leptomeningeal metastasis (6.3% vs. 10.9%, *p* < 0.001). Asymptomatic patients underwent less intensive BM therapy but had a longer median overall survival (statistically significant for a cohort of HER2-positive patients) compared to symptomatic patients (10.4 vs. 6.9 months, *p* < 0.001). *Conclusions*: These analyses show a trend that asymptomatic patients have less severe metastatic brain disease and despite less intensive local BM therapy still have a better outcome (statistically significant for a cohort of HER2-positive patients) than patients who present with symptomatic BM, although a lead time bias of the earlier diagnosis cannot be ruled out. Our analysis is of clinical relevance in the context of potential trials examining the benefit of early detection and treatment of BM.

## 1. Introduction

The incidence of brain metastases (BM) in breast cancer patients has increased in the last decades and has become a major factor in life expectancy and quality of life for many breast cancer patients [1,2]. The evaluation of the National Cancer Register in Sweden showed that compared with the period 1998–2000, patients diagnosed with a primary breast cancer during 2001–2003 were at a 17% increased risk of being admitted with brain metastases during follow-up, and patients diagnosed in 2004–2006 were at a 44% increased risk [3]. 

The retrospective analysis of Darlix et al. showed that 24.6% of patients with metastatic breast cancer develop BM in the course of the disease [4]. The risk for the development of BM differs between the breast cancer subtypes [5,6,7,8]. The literature review of Lin et al. reports a BM rate of 30–55% in patients with metastatic HER2-positive breast cancer and 25–46% in patients with metastatic triple-negative breast cancer [9]. A median overall survival (OS) of 7.4 months after diagnosis of BM was observed in our previous analysis of the Brain Metastases in Breast Cancer (BMBC) Registry [10]. The survival rate of patients with BM has not improved over the last decades. In fact, analysis of the BMBC Registry has shown shorter survival between the time periods 2010–2015 compared to 2000–2009 (median survival of 5.8 months versus 7.6 months, *p* < 0.0001) [10]. 

The survival rates of patients with BM differ significantly depending on the subtype of breast cancer. Patients with a HER2-positive breast cancer show the highest OS rates (median OS of 11.6 months, 95% CI: 10.0–13.4) in the evaluation of the BMBC Registry, and the lowest survival rates could be shown for patients with triple-negative breast cancer (4.6 months, 95% CI: 3.9–5.4). A similar trend was also observed by further studies [11].

It remains unclear whether early detection of asymptomatic BM in breast cancer patients leads to improved clinical outcomes. Currently, BM screening is not part of the management of patients with breast cancer [12,13,14], although brain MRI screening is part of the clinical routine for the management of patients with lung cancer [15,16], which also has a high propensity to metastasize in the brain [17,18,19,20]. 

The aim of this retrospective analysis was to characterize the cohort of breast cancer patients with asymptomatic BM at diagnosis as well as to compare the OS data of patients with and without neurological symptoms of BM. Thus, we intended to assess the possible potential benefit of early detection of BM and set up a rationale for the design of prospective trials.

## 2. Results

### 2.1. Characteristics of Patients with Asymptomatic BM vs. Patients with Neurological Symptoms of BM

A total of 2589 patients were included in the analysis; 2009 had documented neurological symptoms, which would raise the suspicion of BM, and 580 did not have those neurological symptoms. The following symptoms were considered as neurological symptoms indicative or highly suggestive of BMs: headache; persistent nausea or vomiting; visual disturbance; seizure; and motor deficit/changes in motor function or coordination, mental health, or psychological disturbance. 

In the overall cohort, the median age at diagnosis of BM was 57.0 years. In total, 46.4% of the patients (*n* = 1062) had a HER2-positive, 22.8% (*n* = 522) a triple-negative, and 32.1% (*n* = 750) a luminal-A-like/luminal B-like primary breast cancer (defined as estrogen receptor (ER) and/or progesterone receptor (PR) positive, HER2-negative). The majority of the patients presented with a good performance status at the diagnosis of BM (Karnofsky index ≥80%: *n* = 655, 59%). Approximately one third of the patients (30.9%) had one BM at diagnosis, 42.9% of the patients had multiple BMs (≥4). Leptomeningeal metastasis was diagnosed in 9.9% (*n* = 252) of the patients. The majority (*n* = 2139, 82.7%) had extracranial metastases at the time of BM diagnosis. Detailed information of the patients’ characteristics is presented in Table 1 and Table 2. 

After diagnosis of BM, 49.9% of the patients were treated with chemotherapy (49.9% in the asymptomatic patients group and 49.9% in the symptomatic patients group), 13.9% of the patients received endocrine therapy (13.6% in the group of asymptomatic and 14.1% in the group of symptomatic patients), and 36.1% of the patients received a HER2-targeted therapy (36.5% in the group of asymptomatic and 36.0% in the group of symptomatic patients (detailed information about the systemic treatment regimens is shown in Table 3). 

Local BM treatment consisted of surgery alone for 6.5% of all patients (4.4% of asymptomatic and 7.1% of symptomatic patients), cranial radiotherapy alone for 68.0% of all patients (74.7% of asymptomatic and 66.1% of symptomatic patients), or a combination of surgery and cranial radiotherapy for 25.5% of patients (21% of asymptomatic and 26.9% of symptomatic patients, Table 2).

Univariate analysis showed that patients with asymptomatic BM were slightly younger at diagnosis of BM than symptomatic patients (median age 55.5 vs. 57.0 years, *p* = 0.01) and at diagnosis of primary breast cancer (median age 51.0 vs. 52.0, *p* = 0.042), and showed a lower tumor grading of the primary tumor (G1 3.1% vs. 1.4%, *p* = 0.015). Asymptomatic patients had a better performance status at BM diagnosis (Karnofsky index 80–100% 68.4% vs. 57%, *p* < 0.001), a lower number of BMs (one BM 34.1% vs. 30.1%, *p* = 0.027, ≥4 BM: 37.7% vs. 44.4%), a slightly smaller diameter of BM (median: 1.5 vs. 2.2 cm, *p* < 0.001), less frequent leptomeningeal metastasis (6.3% vs. 10.9%, *p* < 0.001), and went on to have less intensive BM therapy (combined surgery and radiotherapy: 21% vs. 26.9%, *p* = 0.001). 

Furthermore, patients with asymptomatic BM had significantly more extracranial metastases at the time of BM diagnosis compared to patients with symptomatic BM (86.7% vs. 81.5%, *p* = 0.003). The most common location of extracranial metastases in patients with asymptomatic BM were bone (54.0%), lung (39.0%), and liver (37.2%). Whereas, the location of extracranial metastases in symptomatic BM patients was bone (43.2%), lung (36.1%), and liver (34.6%). The breast cancer subtype did not differ significantly between the two groups (*p* = 0.195).

Because of the retrospective and multicenter design of our subanalysis, the missing data could not be avoided. The most missing data is recorded for the variables performance status (*n* = 1478), diameter of BM at diagnosis (*n* = 729), type of radiotherapy, local BM therapy (*n* = 564 and *n* = 424), and tumor grading (*n* = 273).

In a multivariate logistic regression analysis with 705 available patients, patients with a lower performance status were significantly more symptomatic than patients with a performance status of 100% (Karnofsky index 80–90%: OR = 5.38, 95%-CI: 3.09–9.36; 60–70%: OR = 6.44, 95%-CI: 3.32–12.5; 10–50%: 14.0, 95%-CI: 3.96–49.7; *p* < 0.001). Furthermore, patients with more than four BM were more frequently symptomatic than asymptomatic (OR = 2.01, 95%-CI: 1.12–3.61; *p* = 0.019). 

### 2.2. Overall Survival of Patients with Asymptomatic vs. Symptomatic BMs

**Figure 1:**
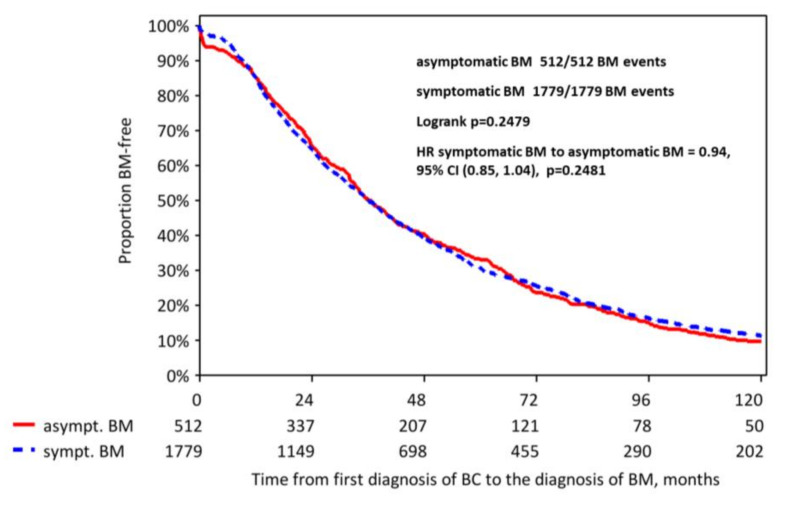
Kaplan-Meier curves for the time from the diagnosis of breast cancer to the diagnosis of BM.

The median time from diagnosis of breast cancer to diagnosis of BM was 36.4 months for patients with asymptomatic BM and 36.0 months for symptomatic patients (*p* = 0.248; Figure 1). 

**Figure 2:**
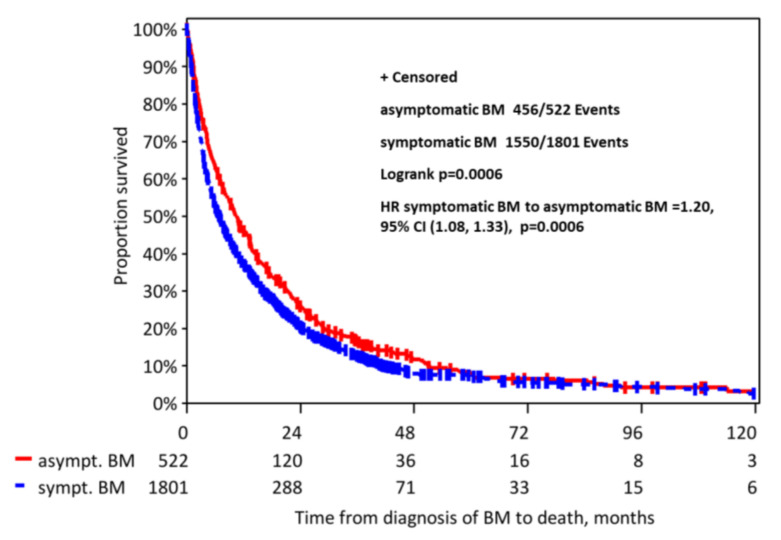
Kaplan-Meier curve for the time from first diagnosis of BM to death due to any reason.

Patients with asymptomatic BM had a longer median overall survival compared to symptomatic patients (10.4 vs. 6.9 months, *p* < 0.001, Figure 2).

When excluding patients with leptomeningeal metastasis from the analysis, the survival difference was less but still significant, with a median OS of asymptomatic patients of 10.4 months (95% CI 8.97–12.8) vs. 7.8 months (95% CI 7.10–8.67) for symptomatic patients (*p* = 0.012).

**Figure 3:**
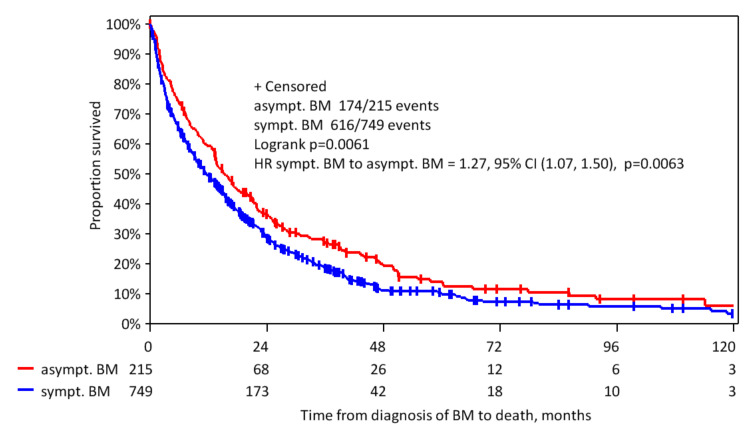
Kaplan-Meier curve for the time from first diagnosis of BM to death due to any reason among HER2-positive patients.

Furthermore, an analysis of OS rates in different tumor subtypes was performed (Table 4). For HER2-positive patients, a statistically significant difference between the OS of asymptomatic and symptomatic patients could be observed (Figure 3, median OS 15.2 vs. 11.5 months, *p* = 0.006). The median OS for patients with TNBC and luminal A- and B-like patients was higher for asymptomatic patients compared to patients with neurologic complaints at BM diagnosis, though the level did not reach statistical significance (*p* = 0.16 and 0.19, respectively). 

The most common cause of death in asymptomatic patients was BM and extracranial metastases (32%), followed by BM only (25.9%). Most of the symptomatic patients (33.1%) died of BM, followed by brain and extracranial metastases (29.1%). Detailed information concerning the causes of death is presented in Table 5. 

### 2.3. Neurologic Symptoms in Different Biological Subtypes

The following neurological symptoms were included in this analysis: seizure, nausea/vomiting, motor deficit, headache, visual disturbance, and mental health or psychological disturbance (Table 6). 

The most frequently documented neurological symptoms were changes in motor function or coordination, with 43.7% (*n* = 228) of TNBC, 40.3% (*n* = 302) of luminal A- and B-like, and 41% (*n* = 435) of HER2-positive patients experiencing these symptoms at diagnosis. About one quarter of the patients (26.0%) complained about headache at presentation of BM. The evaluation of the neurological complaints caused by BM showed a statistically significant difference among the different subtypes concerning the complaints headache, nausea/vomiting, and seizure. The highest rate of seizure could be detected in patients with the luminal A- and B-like subtype (14.8%, *n* = 111), while the lowest prevalence of seizure was detected in patients with TNBC breast cancer (8.6%, *n* = 45). The highest rate of headache could be detected in patients with triple-negative breast cancer (28.3%, *n* = 148); patients with the luminal A- and B-like subtype had the lowest rate of headache (22.1%, *n* = 166). Nausea/vomiting was most frequently documented by patients with triple-negative breast cancer (25.7%, *n* = 134); among luminal A and B-like patients, about 19.1% (*n* = 143) of patients experienced these complaints. 

## 3. Discussion

Our analysis of 2589 patients with BM of breast cancer shows that patients with asymptomatic BM have lower severity of metastatic disease in the brain and have a better outcome despite a less intense local BM therapy compared to symptomatic patients. We additionally evaluated the OS in the following subtypes of breast cancer: HER2-positive, triple-negative, and luminal A- and B-like. Patients without neurological symptoms at BM diagnosis showed a better OS in all breast cancer subtypes: For HER-2 positive, the difference was statistically significant, while for triple-negative and luminal A- and B-like patients, statistical significance was not reached. The limited number of patients in this subgroup analysis must be considered by the interpretation of the results.

Our results correspond to previously published analyses from smaller cohorts. Maurer et al. [21] evaluated 30 asymptomatic HER2-positive breast cancer patients with BM. A better OS was observed for patients with asymptomatic BM in comparison to patients with symptomatic BM (hazard ratio 0.49, 95% CI 0.25–0.94). A significantly higher rate of whole-brain radiotherapy only treatment (63.7% vs. 36.7%, *p* = 0.02) was used in the group of patients with symptomatic BM vs. patients without neurological complaints. The rate of SRS treatment was significantly higher in the group of asymptomatic patients (40% vs. 15.9%, *p* = 0.02). Due to a smaller cohort size with the HER2-positive subtype only, this treatment data is not completely comparable with our analysis. Nonetheless the results match the observation in our cohort that patients without neurological symptoms of BM had less intensive local BM therapy. Maurer et al. report a noticeably higher rate of asymptomatic patients with BM compared to our cohort, with 40.5% (30 of 74 patients with BM) not having neurological complaints. The reason for this high prevalence of asymptomatic patients could lie in a selection bias. According to the authors, the decision for screening for BM in case of metastatic extracranial disease was at the treating physician’s discretion and did not follow any guidelines.

Morikawa et al. [22] analyzed 100 HER2-positive breast cancer patients with BM. The investigators described that the absence of neurologic symptoms at BM diagnosis was significantly associated with fewer BM lesions, decreased use of whole-brain radiotherapy, and longer survival (multivariate hazard ratio 3.69; 95% CI 1.69–8.07). The rate of asymptomatic patients in this study corresponds to the results of our analysis. In total, 22.4% (580 of 2589 patients) did not have neurologic symptoms at the time of BM diagnosis in our cohort. Morikawa et al. reported about 23% of asymptomatic patients in their cohort of 100 patients with BM of HER2-positive breast cancer.

Both investigating groups support the planning of prospective trials evaluating the role of BM screening, especially in patients with HER2-positive metastatic breast cancer. 

A retrospective analysis by Niikura et al. [11] of 1256 patients with BM of breast cancer including all subtypes of whom 262 (20.9%) were asymptomatic neurologically revealed a longer OS in patients with asymptomatic BM vs. patients with neurologic symptoms of BM (hazard ratio 0.81, 95% CI 0.69–0.94, *p* = 0.006). The difference between symptomatic and asymptomatic patients was also found in the multivariate analysis considering other prognostic factors, such as time to BM, number of BM, tumor subtype, and grading. No detailed comparison of patient’s characteristics with and without neurological symptoms was published. 

The HER2CLIMB Trial [23] examining the tyrosine kinase inhibitor tucatinib in addition to standard therapy allowed the inclusion of patients with stable BM. The encouraging results concerning the decreased intracranial progression rate, increased OS, as well as reduced risk of death under treatment with the novel compound tucatinib indicates a potential rationale for BM early detection strategies since patients might benefit more from specific therapies. 

It is important to note, when considering the better OS rate in asymptomatic patients in our cohort, that many of the asymptomatic patients were diagnosed because of scans undertaken for staging purposes as part of clinical trials. This cohort of patients tends to have a better initial performance status when compared with patients with neurological symptoms of BM or who were not so clinically well and would not be included in those clinical trials. This fact may have influenced the analyzed OS times. Furthermore, a better performance status of asymptomatic patients may be related to an absence of neurological complaints. The exact percentage of patients who underwent brain imaging as a part of a clinical trial cannot be captured due to the retrospective multicenter design of this subanalysis.

Our evaluation of the neurological complaints caused by BM showed similar results in the subtypes of BC (TNBC, HER2-positive, luminal A- and luminal B-like). The statistical comparison showed significant differences among the breast cancer subtypes concerning the following neurologic complaints: seizure, nausea/vomiting, and headache. The highest rate of seizures was detected in luminal A- and B-like patients. Several risk factors for the development of seizures in patients with BM are described in the literature: For example, patients with a single BM, detection of tumoral hemorrhage, or supratentorial BM are at a higher risk [24]. Our previous analysis of the BMBC Registry showed that patients with positive estrogen or progesterone status have a lower number of BMs at diagnosis [25]. Thus, possibly a lower number of BMs in luminal-like patients could explain the higher probability of seizures. The lower number of BMs at diagnosis in hormone receptor-positive patients could be a possible explanation for the significant lower rate of headache and nausea in luminal A- and B-like patients. Further research must be performed to confirm these observations.

The strength of our analysis is the large sample size. To our knowledge, we analyzed the largest cohort of patients with asymptomatic BM of breast cancer. Moreover, we analyzed a homogeneous cohort of patients with BM of breast cancer and an absence of other malignant diseases. The limitation of the analysis is that a lead time bias of the earlier diagnosis of BM cannot be ruled out. A further limitation concerning the power of the multivariate analysis must be considered: Because of the missing data of performance status, multivariate logistic regression analysis could only be performed for 705 patients. The reason for a significant number of missing data is the retrospective and multicenter design of our subanalysis. An important characteristic that may have influenced the overall survival rates and performance status of patients is the existence of leptomeningeal metastases. Missing values for this parameter could be detected in 22 patients with asymptomatic BM and 23 patients with symptomatic BM. 

Taking into account the retrospective design of this subanalysis, we cannot recommend a change in clinical practice concerning the management of patients with breast cancer without neurological complaints. The BM screening approach cannot be justified on the basis of our results. International guidelines have recommended no BM screening in breast cancer patients without neurological complaints, but a broad indication for brain imaging should be made if a patient has neurological symptoms related to BM. Our analysis is of clinical relevance in the context of prospective trials examining the benefit of early detection in breast cancer patients with a high risk for BM development. Considering the low time-dependent incidence of BM in early breast cancer patients [26,27], we suggest a prospective trial evaluating BM screening in patients with metastatic disease. It is estimated that almost a quarter of such patients develop BM [28]. A literature review performed by Komorowski et al. shows that in particular patients with HER2-positive and triple-negative metastatic breast cancer have a high incidence of BM (ranging between 22% and 36% and 15% and 37%, respectively). Further risk factors for the development of BM among (HER positive) patients with metastatic breast cancer are younger age, progress of the metastatic breast cancer, and hormone receptor-negative disease [29]. There is less information available concerning risk factors for developing BM in patients with metastatic TNBC. Morris et al. [30] described a patient cohort with TNBC and BM in a follow-up time period: At the time of BM diagnosis, 68% of the patients had evidence of progression of extracranial disease. The analysis of Dawood et al. [31] showed that even patients with advanced TNBC without evidence of distant metastases (stage III) had a significantly higher risk of developing BM (hazards ratio vs. stage I 3.51; 95% CI 1.85–6.67, *p* = 0.0001). When designing a study evaluating BM early detection strategies, tumor subtype and breast cancer stage might help to identify patients at higher risk for developing BM. Biomarker evaluation for the prediction of BM should be integrated in ongoing studies with the perspective to select patients that are candidates for BM early detection. 

## 4. Materials and Methods 

The clinical data for the analysis was derived from the BMBC Registry. Patients with BM of breast cancer registered until April 2019 were considered for this analysis. The BMBC registry was approved by the ethic committee Hessen, Frankfurt am Main (ethic approval number FF42/2013).

The aim of the analysis was to characterize the patient cohort with BM of breast cancer with vs. without documented neurological symptoms. The following neurological symptoms of BM were assessed in the BMBC registry: seizure, nausea/vomiting, motor deficit, headache, visual disturbance, and mental health/psychological disturbance. The following characteristics for the cohort description were assessed: age at first diagnosis of breast cancer and of BM, number and localization of BM, biological subtype, ECOG/Karnofsky performance status at time of BM diagnosis, existence of leptomeningeal metastasis, maximum diameter of BM at first BM diagnosis, extracranial metastases (ECM) at time of BM diagnosis, brain metastases-free interval (time from the first diagnosis of BC to the diagnosis of BM), extracranial metastases-free survival (time from the first diagnosis of BC to ECM in the subgroup of patients with a diagnosis of BM after diagnosis of ECM), and local or contralateral recurrence-free interval (time from the first diagnosis of BC to local or contralateral recurrence) after diagnosis of breast cancer. Furthermore, we performed a comparison of patients with vs. without neurological symptoms regarding the local treatment of BM, year of diagnosis, diagnostic method of BM, and systemic treatment of breast cancer after the diagnosis of BM. Continuous data were summarized using the number of available data, mean, standard deviation (SD), median, and the minimum and maximum for each group. Categorical and ordinal data were summarized using the number and percentage of patients in each group. Differences in the categorical data between patients with symptomatic and asymptomatic BM data were tested by Chi-square or Fisher’s exact test. Differences in the continuous variables between the two patient groups were tested non-parametrically using the Wilcoxon–Mann–Whitney test. For the analysis of the time intervals between the first diagnosis of BC to local or contralateral recurrence, cumulative incidence functions were plotted with death as a competing risk. Differences in the time intervals between symptomatic and asymptomatic patients were tested by Gray’s test. 

A multivariate logistic regression was performed for symptomatic vs. asymptomatic BM. The odds ratios with the corresponding 95% confidence intervals were reported. 

A further objective was to characterize the cohort of patients with neurological symptoms (seizure, nausea/vomiting, motor deficit, headache, impaired vision, mental health/ psychological disturbance) according to the biological subtype of the initial breast cancer. Biological subtype was defined as HER2-positive, TNBC (ER-, PR-negative, and HER2-negative) and luminal A- and B-like (luminal A- and B-like subtypes were evaluated together (ER and/or PR+, HER2-) because of the missing values of tumor grading causing an issue in the strict distinction of luminal A-like and luminal B-like patients). 

Furthermore, we performed an analysis of the OS for the patients with and without neurological symptoms of BM. OS was defined as the time interval from the first diagnosis of BM to death due to any reason. Kapla–Meier curves and the median OS time with the corresponding 95% confidence interval were determined. Differences in the survival curves were tested by the log-rank test. Additionally, an OS analysis in the different breast cancer subtypes (HER2-positive, triple-negative breast cancer, luminal A- and B-like) was performed.

All reported p-values were two-sided, and the significance level was set to 0.05. Confidence intervals symmetrically cover 95%. Adjustment for multiple testing was not planned. 

The data was analyzed using SAS^®^ (Statistical Analysis Software) version 9.4 with SAS Enterprise Guide Version 7.1 on Microsoft Windows 10 Enterprise. 

## 5. Conclusions

Our analyses indicate that asymptomatic patients show a trend for a less severe metastatic disease in the brain and for a better outcome (statistically significant for a cohort of HER2-positive patients) despite a less intense local BM therapy compared to symptomatic patients. Although a lead time bias of the earlier diagnosis cannot be ruled out, this analysis is of potential clinical relevance in the context of potential trials examining the benefit of early detection of BM. 

## Figures and Tables

**Table 1:** Patients’ characteristics, continuous parameters.

Parameter	Category	Asymptomatic Patients *n* = 580	Symptomatic Patients *n* = 2009	Overall *n* = 2589	*p*-Value
Age at first diagnosis of BC, years	Median	51.0	52.0	52.0	0.042
	Min, Max	22.0, 87.0	20.0, 98.0	20.0, 98.0	
	Missing	0	0	0	
Age at diagnosis of BM, years	Median	55.5	57.0	57.0	0.010
	Min, Max	26.0, 87.0	22.0, 99.0	22.0, 99.0	
	Missing	0	0	0	
Max diameter of BM (cm)	Median	1.5	2.2	2.0	<0.001
	Min, Max	0.0, 22.0	0.0, 85.0	0.0, 85.0	
	Missing	200	529	729	

**Table 2:** Patients’ characteristics, categorical parameters.

Parameter	Category	Asymptomatic Patients *n* = 580 (%)	Symptomatic Patients *n* = 2009 (%)	Overall *n* = 2589	*p*-Value
Histological tumor type	ductal or ductal-lobular-invasive	415 (72.3)	1461 (73.5)	1876 (73.3)	0.774
	lobular-invasive	43 (7.5)	151 (7.6)	194 (7.6)	
	other	116 (20.2)	375 (18.9)	491 (19.2)	
	missing	6	22	28	
Tumor grading	G1	16 (3.1)	26 (1.4)	42 (1.8)	0.015
	G2	214 (42.1)	713 (39.4)	927 (40.0)	
	G3	278 (54.7)	1069 (59.1)	1347 (58.2)	
	missing	72	201	273	
Biological subtype	TNBC	103 (19.5)	419 (23.2)	522 (22.4)	0.195
	Luminal A-like and Luminal B-like	178 (33.7)	572 (31.7)	750 (32.1)	
	Her2+	247 (46.8)	815 (45.1)	1062 (45.5)	
	missing	52	203	255	
Karnofsky index at diagnosis of BM	100%	60 (31.6)	94 (10.2)	154 (13.9)	<0.001
	80–90%	70 (36.8)	431 (46.8)	501 (45.1)	
	60–70%	46 (24.2)	276 (30.0)	322 (29.0)	
	40–50%	13 (6.8)	90 (9.8)	103 (9.3)	
	10–30%	1 (0.5)	30 (3.3)	31 (2.8)	
	missing	390	1088	1478	
Number of BM	1	170 (34.1)	548 (30.1)	718 (30.9)	0.027
	2–3	141 (28.3)	466 (25.6)	607 (26.1)	
	≥4	188 (37.7)	809 (44.4)	997 (42.9)	
	missing	81	186	267	
Diagnostic method to detect BM	only clinical	19 (3.5)	57 (2.9)	76 (3.0)	<0.001
	CT ^1^	108 (20.1)	425 (21.5)	533 (21.2)	
	MRI ^1^	382 (71.0)	1283 (64.8)	1665 (66.1)	
	CT and MRI^1^	29 (5.4)	215 (10.9)	244 (9.7)	
	missing	42	29	71	
Local treatment of BM	Surgery only	21 (4.4)	119 (7.1)	140 (6.5)	0.001
	Radiotherapy only	360 (74.7)	1112 (66.1)	1472 (68.0)	
	Surgery and radiotherapy	101 (21.0)	452 (26.9)	553 (25.5)	
	missing	98	326	424	
Type of local radiotherapy of BM	Whole brain radiotherapy only	349 (75.7)	1265 (80.9)	1614 (79.7)	<0.001
	Stereotactic therapy only	52 (11.3)	182 (11.6)	234 (11.6)	
	Whole brain radiotherapy and stereotactic radiotherapy	33 (7.2)	88 (5.6)	121 (6.0)	
	Type of radiotherapy unknown	27 (5.9)	29 (1.9)	56 (2.8)	
	missing	119	445	564	
Extracranial metastases at time of BM diagnosis	yes	502 (86.7)	1637 (81.5)	2139 (82.7)	0.003
	no	77 (13.3)	371 (18.5)	448 (17.3)	
	missing	1	1	2	
Localization of first metastasis if extracranial					
bone metastases	no	267 (46.0)	1141 (56.8)	1408 (54.4)	<0.001
	yes	313 (54.0)	868 (43.2)	1181 (45.6)	
	missing	0	0	0	
liver metastases	no	364 (62.8)	1314 (65.4)	1678 (64.8)	0.256
	yes	216 (37.2)	695 (34.6)	911 (35.2)	
	missing	0	0	0	
lung metastases	no	354 (61.0)	1283 (63.9)	1637 (63.2)	0.222
	yes	226 (39.0)	726 (36.1)	952 (36.8)	
	missing	0	0	0	
skin metastases	no	538 (92.8)	1903 (94.7)	2441 (94.3)	0.084
	yes	42 (7.2)	106 (5.3)	148 (5.7)	
	missing	0	0	0	
other metastases	no	402 (69.3)	1468 (73.1)	1870 (72.2)	0.082
	yes	178 (30.7)	541 (26.9)	719 (27.8)	
	missing	0	0	0	
Existence of leptomeningeal metastasis	no	523 (93.7)	1769 (89.1)	2292 (90.1)	<0.001
	yes	35 (6.3)	217 (10.9)	252 (9.9)	
	missing	22	23	45	
Year of BM diagnosis	<2010	205 (35.3)	583 (29.0)	788 (30.4)	0.004
	≥2010	375 (64.7)	1426 (71.0)	1801 (69.6)	
	missing	0	0	0	

^1^ with or without clinical symptoms.

**Table 3:** Breast cancer treatment after BM diagnosis (systemic treatment, endocrine therapy, targeted therapy) for patients with and without neurological symptoms.

Treatment Specification	Asymptomatic Patients	Symptomatic Patients	Overall
	*n* (%)	*n* (%)	*n* (%)
Anthracycline	43 (4.95)	107 (4.99)	150 (4.98)
Taxane based	66 (7.59)	117 (5.45)	183 (6.07)
Taxane and Anthracycline	108 (12.43)	289 (13.47)	397 (13.17)
other chemotherapy	217 (24.97)	558 (26.01)	775 (25.71)
Tamoxifen	18 (2.07)	36 (1.68)	54 (1.79)
Aromatase inhibitor	63 (7.25)	172 (8.02)	235 (7.80)
GnRH-analoga	9 (1.04)	19 (0.89)	28 (0.93)
other hormone therapy	28 (3.22)	75 (3.50)	103 (3.42)
Trastuzumab	85 (9.78)	222 (10.35)	307 (10.19)
Trastuzumab + Pertuzumab	9 (1.04)	37 (1.72)	46 (1.53)
Lapatinib	64 (7.36)	172 (8.02)	236 (7.83)
T-DM1	34 (3.91)	79 (3.68)	113 (3.75)
Everolimus	6 (0.69)	25 (1.17)	31 (1.03)
Bisphosphonates	66 (7.59)	112 (5.22)	178 (5.91)
Denosumab	26 (2.99)	62 (2.89)	88 (2.92)
Bevacizumab	27 (3.11)	59 (2.75)	86 (2.85)
other targeted therapy	--	4 (0.19)	4 (0.13)

**Table 4:** Median OS in different groups of biological subtypes for symptomatic and asymptomatic patients.

Biological Subtype	Neurological Symptoms	Median OS (95% CI)
TNBC	no	6.5 (4.7, 9.0)
TNBC	yes	4.1 (3.4, 4.8)
Luminal A and Luminal B-like	no	8.5 (5.5,10.9)
Luminal A and Luminal B-like	yes	5.7 (4.9, 6.8)
HER2+	no	15.2 (13.3,19.8)
HER2+	yes	11.5 (10.0,13.7)

**Table 5:** Causes of death in patients with asymptomatic and symptomatic BM.

Causes of Death	Asymptomatic Patients *n* = 456 (%)	Symptomatic Patients *n* = 1550 (%)	Overall *n* = 2006 (%)	*p*-Value
Brain metastases	118 (25.9)	513 (33.1)	631 (31.5)	0.091
Extracranial metastases	72 (15.8)	229 (14.8)	301 (15.0)	
Brain and extracranial metastases	146 (32.0)	451 (29.1)	597 (29.8)	
Not known but tumor related	27 (5.9)	82 (5.3)	109 (5.4)	
Not tumor related	25 (5.5)	87 (5.6)	112 (5.6)	
Not known	68 (14.9)	188 (12.1)	256 (12.8)	
Missing	0	0	0	

**Table 6:** Distribution of the neurological symptoms according to the biological subtypes.

Symptom	TNBC		Luminal A/Luminal B		HER2+		Overall		
	*n*	%	*n*	%	*n*	%	*n*	%	*p*-Value *
Headache									
no	374	71.65	584	77.87	775	72.98	1733	74.25	0.019
yes	148	28.35	166	22.13	287	27.02	601	25.75	
Visual disturbance									
no	446	85.44	649	86.53	912	85.88	2007	85.99	0.850
yes	76	14.56	101	13.47	150	14.12	327	14.01	
Mental health or psychological disturbance									
no	442	84.67	622	82.93	906	85.31	1970	84.40	0.382
yes	80	15.33	128	17.07	156	14.69	364	15.60	
Change in motor function or coordination/motor deficit									
no	294	56.32	448	59.73	627	59.04	1369	58.65	0.450
yes	228	43.68	302	40.27	435	40.96	965	41.35	
Nausea/vomiting									
no	388	74.33	607	80.93	828	77.97	1823	78.11	0.020
yes	134	25.67	143	19.07	234	22.03	511	21.89	
Seizure									
no	477	91.38	639	85.20	926	87.19	2042	87.49	0.004
yes	45	8.62	111	14.80	136	12.81	292	12.51	
none									
no	451	86.40	620	82.67	899	84.65	1970	84.40	0.188
yes	71	13.60	130	17.33	163	15.35	364	15.60	

* Chi-squared test: the *p*-values are to be understood descriptively; they were not corrected for multiple comparisons.

## References

[1] Meattini I., Andratschke N. (2020). Challenges in the treatment of breast cancer brain metastases: Evidence, unresolved questions, and a practical algorithm. Clin. Transl. Oncol..

[2] Witzel I., Oliveira-Ferrer L. (2016). Breast cancer brain metastases: Biology and new clinical perspectives. Breast Cancer Res..

[3] Fisk G., Svensson T. (2012). Incidence and time trends of brain metastases admissions among breast cancer patients in Sweden. Br. J. Cancer.

[4] Darlix A., Louvel G. (2019). Impact of breast cancer molecular subtypes on the incidence, kinetics and prognosis of central nervous system metastases in a large multicentre real-life cohort. Br. J. Cancer.

[5] Soni A., Ren Z. (2015). Breast cancer subtypes predispose the site of distant metastases. Am. J. Clin. Pathol..

[6] Kennecke H., Yerushalmi R. (2010). Metastatic behavior of breast cancer subtypes. J. Clin. Oncol..

[7] Sihto H., Lundin J. (2011). Breast cancer biological subtypes and protein expression predict for the preferential distant metastasis sites: A nationwide cohort study. Breast Cancer Res..

[8] Smid M., Wang Y. (2008). Subtypes of breast cancer show preferential site of relapse. Cancer Res..

[9] Lin N.U., Amiri-Kordestani L. (2013). CNS metastases in breast cancer: Old challenge, new frontiers. Clin. Cancer Res..

[10] Witzel I., Laakmann E. (2018). Treatment and outcomes of patients in the Brain Metastases in Breast Cancer Network Registry. Eur. J. Cancer.

[11] Niikura N., Hayashi N. (2014). Treatment outcomes and prognostic factors for patients with brain metastases from breast cancer of each subtype: A multicenter retrospective analysis. Breast Cancer Res. Treat..

[12] Ramakrishna N., Temin S. (2018). Recommendations on disease management for patients with advanced human epidermal growth factor receptor 2—Positive breast cancer and brain metastases: ASCO clinical practice guideline update. J. Clin. Oncol..

[13] NCCN Clinical Practice Guidelines in Oncology. Breast Cancer. Version 3.2020. https://www.nccn.org/store/login/login.aspx?ReturnURL=https://www.nccn.org/professionals/physician_gls/pdf/breast.pdf.

[14] Ditsch N., Untch M. (2020). AGO recommendations for the diagnosis and treatment of patients with locally advanced and metastatic breast cancer: Update 2020. Breast Care.

[15] NCCN Guidelines Small Cell Lung Cancer. Version 2.2020. https://www2.tri-kobe.org/nccn/guideline/lung/english/small.pdf.

[16] NCCN Guidelines Non-Small Cell Lung Cancer. Version 3.2020. https://www2.tri-kobe.org/nccn/guideline/lung/english/non_small.pdf.

[17] Schouten L.J., Rutten J. (2002). Incidence of brain metastases in a cohort of patients with carcinoma of the breast, colon, kidney, and lung and melanoma. Cancer.

[18] Goncalves P.H., Peterson S. (2016). Risk of brain metastases in patients with non-metastatic lung cancer: Analysis of the metropolitan detroit Surveillance, Epidemiology, and End Results (SEER) data. Cancer.

[19] Barnholtz-Sloan J.S., Sloan A.E. (2004). Incidence proportions of brain metastases in patients diagnosed (1973 to 2001) in the metropolitan Detroit cancer surveillance system. J. Clin. Oncol..

[20] Chamberlain M.C., Baik C.S. (2017). Systemic therapy of brain metastases: Non-small cell lung cancer, breast cancer, and melanoma. Neuro Oncol..

[21] Maurer C., Tulpin L. (2018). Risk factors for the development of brain metastases in patients with HER2-positive breast cancer. ESMO Open.

[22] Morikawa A., Wang R. (2018). Characteristics and prognostic factors for patients with HER2-overexpressing breast cancer and brain metastases in the era of HER2-targeted therapy: An argument for earlier detection. Clin. Breast Cancer.

[23] Lin N.U., Borges V. (2020). Intracranial efficacy and survival with tucatinib plus trastuzumab and capecitabine for previously treated HER2-positive breast cancer with brain metastases in the HER2CLIMB trial. J. Clin. Oncol..

[24] Wolpert F., Lareida A. (2019). Risk factors for the development of epilepsy in patients with brain metastases. Neuro Oncol..

[25] Laakmann E., Witzel I. (2016). Radiological patterns of brain metastases in breast cancer patients: A subproject of the German Brain Metastases in Breast Cancer (BMBC) registry. Int. J. Mol. Sci..

[26] Laakmann E., Witzel I. (2019). Development of central nervous system metastases as a first site of metastatic disease in breast cancer patients treated in the neoadjuvant trials GeparQuinto and GeparSixto. Breast Cancer Res..

[27] Komorowski A.S., Warner E. (2020). Incidence of Brain metastases in nonmetastatic and metastatic breast cancer: Is there a role for screening?. Clin. Breast Cancer.

[28] Pasquier D., Darlix A. (2020). Treatment and outcomes in patients with central nervous system metastases from breast cancer in the real-life ESME MBC cohort. Eur. J. Cancer.

[29] Hurvitz S.A., O’Shaughnessy J. (2019). Central nervous system metastasis in patients with HER2-positive metastatic breast cancer: Patient characteristics, treatment, and survival from SystHERs. Clin. Cancer Res..

[30] Morris P.G., Murphy C.G. (2012). Limited overall survival in patients with brain metastases from triple negative breast cancer. Breast J..

[31] Dawood S., Lei X. (2012). Incidence of brain metastases as a first site of recurrence among women with triple receptor-negative breast cancer. Cancer.

